# Randomized, Placebo-Controlled, Double-Blind Pilot Study of D-Cycloserine in Chronic Stroke

**DOI:** 10.1155/2015/534239

**Published:** 2015-10-26

**Authors:** Andrew J. Butler, Justiss Kallos, Stephen N. Housley, Michelle C. LaPlaca, Stephen F. Traynelis, Steven L. Wolf

**Affiliations:** ^1^Department of Physical Therapy, School of Nursing and Health Professions, Georgia State University, Atlanta, GA 30303, USA; ^2^Atlanta Veterans Affairs Medical Center, Rehabilitation Research and Development Center of Excellence for Visual and Neurocognitive Rehabilitation, Decatur, GA 30033, USA; ^3^Wallace H. Coulter Department of Biomedical Engineering, Georgia Institute of Technology, Atlanta, GA 30332, USA; ^4^Department of Pharmacology, Emory University, Atlanta, GA 30332, USA; ^5^Division of Physical Therapy, Department of Rehabilitation Medicine, Emory University School of Medicine, USA

## Abstract

Stroke is a leading cause of death and disability in the USA. Up to 60% of patients do not fully recover despite intensive physical therapy treatment. N-Methyl-D-aspartate receptors (NMDA-R) have been shown to play a role in synaptic plasticity when activated. D-Cycloserine promotes NMDA receptor function by binding to receptors with unoccupied glycine sites. These receptors are involved in learning and memory. We hypothesized that D-cycloserine, when combined with robotic-assisted physiotherapy (RAP), would result in greater gains compared with placebo + RAP in stroke survivors. Participants (*n* = 14) were randomized to D-cycloserine plus RAP or placebo plus RAP. Functional, cognitive, and quality-of-life measures were used to assess recovery. There was significant improvement in grip strength of the affected hand within both groups from baseline to 3 weeks (95% confidence interval for mean change, 3.95 ± 2.96 to 4.90 ± 3.56 N for D-cycloserine and 5.72 ± 3.98 to 8.44 ± 4.90 N for control). SIS mood domain showed improvement for both groups (95% confidence interval for mean change, 72.6 ± 16.3 to 82.9 ± 10.9 for D-cycloserine and 82.9 ± 13.5 to 90.3 ± 9.9 for control). This preliminary study does not provide evidence that D-cycloserine can provide greater gains in learning compared with placebo for stroke survivors.

## 1. Introduction

An estimated 750,000 Americans suffer a stroke annually, incurring estimated costs related to their care of approximately $56.8 billion [1]. Stroke is a leading cause of serious long-term disability and the long-term effects of stroke affect an estimated 6.4 million Americans [2]. In addition, 50–60% of stroke survivors exhibit some degree of motor impairment and require at least partial assistance in activities of daily living [3, 4]. The most common impairments that limit functional status after stroke are upper limb motor dysfunction, specifically hand function [5] and gait [6]. The burden of stroke-related disability is predicted to increase in the coming decades in proportion to the expansion of the elderly population [7]. Although stroke case fatality has declined, stroke incidence has not, leading to rising numbers of stroke survivors.

After ischemic damage to motor areas of the brain, patients experience some degree of spontaneous recovery [8, 9], which has increased since the advent of interventions implemented in the acute period after stroke: notably, use of tissue plasminogen activator (TPA) to dissolve blood clots. Of those stroke survivors who do not spontaneously recover, more than 50% will experience motor deficits [10]. One of the most common and enduring impairments following stroke is loss of arm and hand function and considerable time and resources are often spent to restore upper limb movement [5]. Recovery can often take many months and is not always effective [8, 11, 12].

Associated recovery of motor function in the affected upper limb can be incomplete in up to 60% of stroke survivors despite intensive rehabilitation programs [13]. The current standard for intense physical therapy (PT) most commonly consists of neurofacilitation techniques and/or task-specific training [14]. Implementation of intensive physical therapy, such as constraint-induced movement therapy (CIMT), poses a variety of logistical challenges, as it requires substantial time for setup and administration of the therapy in a time-limited environment [3]. Neurotherapy methods also involve the use of extensive human resources to provide several hours of treatment and may suffer from lack of treatment fidelity due to variability in treatment techniques between therapists [3, 4, 15]. Labor-intensive and costly therapy methods are critical barriers to achieving optimal functional outcomes in stroke survivors with motor impairments.

There is a great need to find new ways to enhance the effectiveness of upper limb rehabilitation in patients following stroke. Activation of N-methyl-D-aspartate receptors (NMDA-R) is important for inducing various forms of synaptic plasticity [16]. D-Cycloserine is an established antibiotic drug for the chronic treatment of tuberculosis in humans. Application of D-cycloserine can enhance certain models of plasticity, including long-term potentiation [17], and has recently been investigated as an augmentation therapy for psychological treatment procedures [18]. Such improvements might occur because D-cycloserine promotes NMDA receptor functions by binding to receptors with unoccupied glycine sites and perhaps by selectively enhancing the activity of NMDA-R [19, 20], which are critically involved in learning and memory.

Therefore, promotion of NMDA receptor function by administration of D-cycloserine at 100 mg may modulate NMDA receptor activity and has been suggested to improve cognition in patients with Alzheimer's disease [21, 22]. Compared to placebo, 50 mg of D-cycloserine has been shown to enhance therapeutic learning from exposure-based cognitive-behavior therapy in patients with anxiety-related disorders [18].

Two studies have tested D-cycloserine as an adjuvant to rehabilitation of stroke-related impairment. Cherry et al. [23] found no significant difference in motor performance on a stability platform balance task (Lafayette Instrument, model 16030L) or a simulated feeding task [24], when a single dose of D-cycloserine (250 mg) was compared to placebo (250 mg) over the course of one training day. Nadeau et al. [25] posited that 50 mg of D-cycloserine combined with constraint-induced movement therapy would increase learning because of its potential of sodium-calcium influx through NMDA-glutamate voltage-gated sodium-calcium channels, which are crucial to learning. The D-cycloserine treatment failed to yield greater learning and retention compared to placebo.

A promising approach to improving upper extremity motor function utilizes repetitive task practice (RTP) and behavioral shaping along with constraint of the less affected limb, known as constraint-induced movement therapy [26]. Two fundamental limitations of CIMT are the time necessary to deliver and oversee training and the excessive time during which the less affected limb must be constrained. RTP, in the context of CIMT, appears to be effective in improving upper extremity motor function of patients with stroke [26]. Alternative approaches, such as robotic-assisted physiotherapy (RAP), have also been investigated as a delivery method to improve upper extremity motor function using repetitive task practice. Recent evidence suggests that improvements in upper extremity motor function, daily task performance, and quality of life are seen during a robotic-assisted physical therapy regimen [27].

Cognition plays an important role in performing motor skills [28]; however, the role of cognition during physical therapy treatment is unclear. Motor learning involves more than storing sensory and motor information that arises as a consequence of movement. Skill learning is highly cognitive, and the cognitive processes that subserve movement must be practiced. D-Cycloserine has been shown to have an effect on procedural learning processes [29]. Procedural motor learning most commonly entails acquiring novel movement patterns, which is also the main objective in rehabilitation of motor deficits after stroke. Deficient procedural motor learning could therefore contribute to incomplete recovery of motor functions in the chronic poststroke phase.

The purposes of this pilot study were to (1) evaluate the safety and tolerance of D-cycloserine + RAP over three weeks of study participation for stroke survivors and (2) evaluate whether D-cycloserine + RAP improved grip strength, Box and Block test (BBT) performance, daily robotic weighted scores, and health related quality-of-life (HRQL) scores, when compared to placebo + RAP over three weeks of study participation for stroke survivors.

## 2. Methods and Materials

### 2.1. Subjects and Study Overview

Fourteen participants (3 males; 55.8 ± 13.1 years of age; time after stroke 391.2 ± 252.1 days) with chronic (greater than 3 months) ischemic stroke were selected to participate in this double-blind, randomized controlled trial.

The main entry criteria were a single unilateral ischemic stroke 3 to 48 months prior to the study, age 18 to 95 years, at least 10° of active wrist extension, at least 10° of thumb abduction/extension, and at least 10° of extension in at least two additional digits [30]. Potential participants were excluded if they scored less than 24 on the Minimental State Examination (MMSE) [31] or if physician-determined major medical problems could interfere with participation. Additional exclusion criteria were previously clinically documented stroke; excessive pain in any joint of the paretic extremity; a substantial decrease in alertness, language reception, or attention; pregnancy or lactation; advanced systemic medical disease; coexistent major neurologic or psychiatric disease; orthostatic hypotension; concurrent use of drugs known to interfere with the action of D-cycloserine; concurrent enrollment in another stroke recovery investigation; or any contraindication to D-cycloserine. The choice of restricting the time frame of stroke to at least 3 months after stroke was intended to ensure that acute stroke medical issues would have stabilized. The choice of no more than 48 months after stroke was intended to minimize the variance introduced by late poststroke changes, for example, contractures and psychosocial decline.

**Figure 1:**
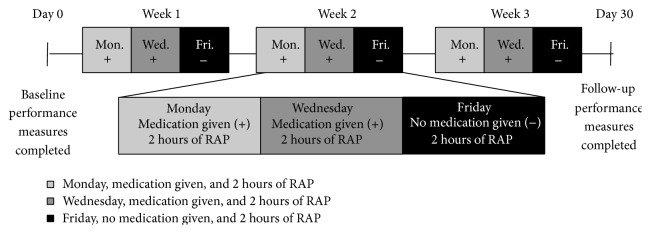
Experimental design and time course of the study. After completing baseline testing, there were 6 visits at which medication (placebo or D-cycloserine at 100 mg dose) was dispensed. Dosing was oral and twice weekly (Monday and Wednesday) throughout the study. The study design included one session per week (Friday) in which the drug was not dispensed. Volunteers received medication immediately preceding the robotic training sessions. Final examination was administered on Day 30. + indicates medication given and − indicates no medication given. RAP: robotic-assisted physiotherapy.

After informed consent was obtained, eligibility was determined and qualified subjects were randomized in a 1 : 1 fashion to either D-cycloserine or placebo in a double-blind manner. Computer-generated randomization schedules were generated with envelopes connecting subject identification to treatment arm assignment provided to an unblinded pharmacist. Patients received either drug or placebo before two (Monday and Wednesday) of three treatments each week for three weeks, with concomitant RAP during all treatment sessions (Figure 1). Assessments were performed at baseline (Day 0) and follow-up (Day 30). The robotic device recorded outcomes during the nine training sessions that were used to assess motor outcome. Local institutional review boards approved all procedures.

### 2.2. Therapeutic Intervention

D-Cycloserine is an FDA approved medication that does not require titrating. Infrequent side effects in patients on chronic dosing schedule (who were generally chronically ill with tuberculosis) have included drowsiness, headache, confusion, tremor, vertigo, memory difficulties, paraesthesias, and seizures [32]. There were six visits at which placebo or D-cycloserine at 100 mg dose was dispensed. Dosing was oral and twice weekly (Monday and Wednesday) throughout the study (Figure 1). The study design included one session per week (Friday) in which the drug was not dispensed. The patients received two doses of D-cycloserine (or placebo) per week, reducing chance for tachyphylaxis (i.e., rapidly decreasing response to a drug after administration of a few doses). Furthermore, recent D-cycloserine psychotherapy trials have observed more rapid within-training improvements than those receiving the placebo [18]; therefore, we anticipate that the current dosing regimen allowed any within-training improvements to be captured on Friday's RAP sessions in the absence of drug.

RAP was provided two hours per day (9 days in total), for a total of 18 hours distributed over three consecutive weeks. A standard RAP protocol was used across people using the HandMentor Pro (KMI Inc., Tempe, AZ 85282). Each RAP session began 20 minutes after pill ingestion and focused on wrist/arm therapy. Volunteers were encouraged to exercise at home, but no formal home-exercise routine was prescribed.

The robotic device uses computer-game-like training programs for motor control plus one spasticity reduction program. A study by Wolf et al. [33] contains a complete description of the HandMentor Pro including the system and air muscle assembly, program/training options, performance tables, and daily game activity charts (please refer to Figure  2 in Wolf et al. [33]). The aim of the training programs is to increase active range of motion (AROM) of wrist and finger flexion and extension and improve the accuracy of these actions. The two main training programs used were balloon and thera-pong. The object of the balloon game is to fly a balloon across the ocean while avoiding obstacles. The aim of the thera-pong game is to defeat the opponent in a simulated table tennis game by earning a higher score. The volunteer uses their affected wrist to control an in-game paddle (or balloon) by moving it vertically across the left side of the screen and competes against a computer-controlled opponent.

### 2.3. Behavioral Testing

Bilateral upper extremity motor function was quantitatively assessed using the Box and Block test and hand dynamometry. Both the affected and unaffected sides were tested to allow comparison between sides. Sustained attention was assessed using a test of rapid visual information processing (RVIP) [34, 35]. Information processing speed and episodic and working memory were measured using the Display Enhanced Testing for Concussions and mild traumatic brain injury (mTBI) system (DETECT system, Zenda LLC, Atlanta, GA). Health-related quality of life was assessed by the 8 subscales and overall stroke recovery rating of the Stroke Impact Scale (SIS) version 3.0 [36]. The functional outcome measures and computer-based measures were performed at baseline (Day 0) prior to treatment and again at follow-up (Day 30).

#### 2.3.1. Upper Limb Motor Function

The primary outcome measures used to assess changes in motor performance included Box and Block test [37] and hand-grip strength using a hand dynamometer. The Box and Block test was chosen because it involves dexterous manipulation of objects and voluntary motor control, which has been shown to improve with upper limb recovery following stroke [38]. Hand-grip strength has been shown to correlate with clinical scores of improved recovery [39]. Hand-grip strength was assessed using the whole hand and defined as the average of 3 trials using a calibrated Jamar dynamometer (Jamar Dynamometer, Asimow Engineering Co., Santa Monica, CA), with the elbow flexed to 90° and the forearm in a neutral position.

#### 2.3.2. Sustained Attention and Short-Term Working Memory

Participants were given the rapid visual information processing task (RVIP) [34, 35] which is a continuous performance test lasting 7 minutes during which time participants are required to monitor a continuous stream of digits, presented at the rate of 100 digits per minute, for prespecified digit strings (e.g., 3-5-7, in consecutive order). Participants respond to the target strings by pressing the spacebar on a computer keyboard. Any two sequences were separated by a minimum of 5 and a maximum of 30 digits. Correct detection (“hits”) of target strings can be registered during the last digit of a sequence or in the subsequent 1800 milliseconds. The average latency of correct detection and the number of commission errors (false alarms to nontarget) are also assessed. Correct detection and commission errors are converted to the single detection variable termed target sensitivity (A-prime) [40, 41]. Target sensitivity is an index of perceptual discriminability of target stimulus from noise (scores range from 0 to 1), whereas response bias indicates the tendency to respond regardless of whether a target is present (scores range from −1 to +1). Reaction time was also measured and defined as the time taken to respond to an experimental target.

#### 2.3.3. Cognitive Function

The Display Enhanced Testing for Concussions and mTBI system (DETECT) was used to assess cognitive function. The tests include (1) Complex Attention, (2) Go-No-Go, (3) Selective Reminding Memory Test, and (4) N-Back Working Memory Test [42].

The DETECT test measure included overall accuracy and response times for the simple and complex attention conditions and for N-Back 1 and N-Back 2 conditions. For the Selective Reminding Test, these included the total number of hits and false alarms, as well as reaction times for both the immediate and delayed recall conditions. The accuracy of a test was treated as a continuous variable.

Individual items were designed to be answered with a dichotomous “yes” or “no” response. In addition to simplicity and ease of use for the test taker, “yes and no” response recognition has been validated and is equivalent to forced choice response when examining recognition memory [43]. Performance was scored based upon response type (correct, incorrect, and missing) and response time (to the hundredth of a second). The Selective Reminding and N-Back test items were presented for 2-second intervals. If no response was logged within 2 seconds, the next item was presented. The Go-No-Go and Complex Choice Reaction Time tests were presented for 3 seconds and if no response was logged within 3 seconds, the next item was presented. This approach ensured shortened battery, while challenging the patient to respond quickly, thereby evaluating potentially slow information processing speed as an indicator of cognitive difficulty.

#### 2.3.4. HRQL

The Stroke Impact Scale (SIS), version 3.0, includes 59 items that comprise 8 domains: strength, hand function, combined basic ADLs and instrumental ADLs (ADL/IADL), mobility, memory and thinking, communication, social participation, and emotion and mood regulation. An overall rating of stroke recovery also is included. Each domain contains a general description of the type of question that follows and a statement with a reference to a specific time period (1, 2, or 4 weeks). Respondents score their performance on a 5-point scale (i.e., “no strength” to “a lot of strength”; “never” to “all of the time”). Duncan et al. [36] have shown the SIS to be valid, reliable, and sensitive to change, and other investigators also have concluded that the SIS has good psychometric properties [44]. A 4.5-to-17.8-point change in a domain score may represent a clinically significant change [45].

#### 2.3.5. Depressive Symptoms

Because depression is an important influence on the quality of life of stroke survivors, the Center for Epidemiologic Studies Depression (CES-D) scale was also included to control for depressive symptoms when investigating the association between ARM/RTP and health-related quality-of-life indicators. In the CES-D scale, each item is rated on a scale from 0 to 3; scores range from 0 to 60 with higher scores indicating more depressive symptoms. A score of 16 or above indicates possible depression.

#### 2.3.6. Robotic Weighted Score

The robotic device uses computer-game-like training programs for motor control and one spasticity reduction program. The two main training programs used were balloon and thera-pong. For each cycle of the balloon (see (1a)) or thera-pong (see (1b)) program, the robotic controller generates a weighted score per minute. This score is generated using numerous measurements, including the number of successful flights of the balloon (or rallies in table tennis) completed, the number of flights (or rallies) attempted, the overall duration of the cycle (32.29 ± 2.36 minutes each), and overall score. The score value is calculated based on the number of possible goals (i.e., number of cycles assigned), number of goals achieved, start angle, and difficulty level. Higher levels indicate better performance:

(1a) weighted scoresecond=1+flights completedflights attempted·scoreminutes played,

(1b) weighted scoresecond=1+rallies completedrallies attempted·scoreminutes played.

### 2.4. Statistical Analysis

Statistical analyses included both nonparametric and 2-tailed parametric methods. Baseline characteristics of the two treatment groups were evaluated for differences using *t*-tests, with special attention being paid to the statistical power to evaluate the probability of type II error. Change scores were calculated by subtracting baseline from outcome scores at week 3. Continuous variables were compared by repeated measures ANOVA, followed by* post hoc* ANCOVA testing in order to explore how baseline performance influenced overall recovery measures and whether baseline performance correlated with overall performance on outcome measures of recovery. A significant interaction between time and group would support the hypothesis that DCS facilitates learning. For daily robotic measures, missing data were imputed by carrying the last measured value forward. If data were missing for measures performed at baseline and follow-up, only available values were used for statistical analysis. All analyses were performed with a minimal level of significance set at *α* = 0.05 using SPSS Statistics Version 21.0 (SPSS Inc., Armonk, NY 10504).

#### 2.4.1. Sample Size

Initial power estimates, adapted from Chen et al. [46], anticipated a baseline BBT score of 29.6 ± 13.4 blocks (mean ± SD) and end-of-treatment BBT score of 35.6 blocks for subjects in the D-cycloserine + RAP group versus 33 blocks for those in the placebo + RAP group, suggesting that 14 patients were required in each study arm to achieve 80% power at *α* = 0.05. Data are expressed as mean ± SD unless otherwise stated. A sample size of 14 for each study arm was not feasible given the fiscal limitations inherent in the preliminary study; instead, 7 volunteers were enrolled in each study arm.

#### 2.4.2. UE Motor Function, Cognition, and HRQL

Motor function (BBT and hand-grip strength), cognition (A-prime and DETECT), and HRQL (SIS and CES-D) were analyzed using group (D-cycloserine + RAP, placebo + RAP) by time (pre- and posttreatment) repeated measures ANOVA comparing the average changes over time on independent variables for the two treatment groups. This was followed by a univariate ANCOVA analysis comparing the magnitude of improvement between the treatment groups (D-cycloserine + RAP, placebo + RAP) across time (pre- and posttreatment) with baseline performance as a covariate. To correct for errors associated with multiple comparisons, *p* values were corrected using the false discovery rate method.

The partial Eta squared (*η*_*p*_^2^) effect size statistic (which indicates the proportion of the effect and error variance, i.e., attributable to the effect) was obtained as part of the repeated measures ANOVA and ANCOVA analysis. For repeated measures ANOVA analysis, ≤0.10 is considered a small effect size, 0.25 is a moderate effect size, and ≥0.40 is a large effect size [47]. For ANCOVA analysis, ≤0.01 is a small effect size, 0.07 is a moderate effect size, and ≥0.19 is a large effect size [47].

The odds of being in one group (e.g., success) relative to the odds of being in a different group (e.g., failure) are captured in the odds ratio (OR) statistic. Average SIS scores were also analyzed using a 2 × 2 odds ratio analysis comparing the number of SIS categories that improved on average by clinically significant amounts between the two groups and by collapsing the individual number of positive outcomes (≥10 improvement) across all subjects and comparing the values between the two groups. OR ranges from 0 to *∞*. OR > 1 indicates an increase in odds relative to the reference group. OR < 1 indicates a decrease in odds relative to the reference group. In general, ≤2 is a small effect size, 3 is a moderate effect size, and ≥4 is a large effect size [48]. The Chi square (*χ* ^2^) test was used to examine differences of categorical variables. All Chi square (*χ* ^2^) *p* values reported were corrected using the Yates continuity method. While *χ* ^2^ is a measure of the significance of association between variables, the phi squared (*ϕ* ^2^) value is a measure of the degree of association between the variables. It can be interpreted as an effect size for *χ* ^2^ analysis. In general, *ϕ* ^2^ ≤ 0.04 is a small effect size, 0.25 is a moderate effect size, and ≥0.64 is a large effect size [47].

#### 2.4.3. Robotic Weighted Score

Daily robotic measures were analyzed using planned nested repeated measures ANOVA. ANCOVA was used in a subsequent secondary exploratory analysis to better understand the significant interaction between treatment group and SIS hand domain change score. Testing occurred over three weeks, and nested within each week were three tests (two with medication on Monday and Wednesday and one without on Friday, Figure 1).

## 3. Results

### 3.1. Subjects and Safety

A total of 124 patients were screened of whom 14 (4 men, 10 women; 4 Caucasian, 10 African American) were enrolled. The most common reasons for screening failure were that the patient lacked sufficient wrist, thumb, or digit active range of motion, was >4 years after stroke (*n* = 6), or declined participation (*n* = 7). Of the 14 patients that completed testing, one participant in the placebo + RAP group was excluded from the analyses because Tukey's schematic box plot analysis [49] identified her as an outlier; subsequent analyses were performed on 13 participants (3 men, 10 women; 3 Caucasian, 10 African American).

Overall, at baseline, subjects were well matched across the 2 treatment groups, although more women were enrolled than men (Table 1). The D-cycloserine group consisted of all women, whereas the placebo group consisted of 3 men and 3 women (*p* = 0.033) and had significantly lower unaffected hand-grip strength scores (23.6 ± 6.6 compared with placebo 34.5 ± 5.9, *p* = 0.011). The days after stroke did not differ between treatment groups (*p* = 0.334), nor did MMSE (*p* = 0.673) and CES-D (*p* = 0.185) scores at baseline. An independent-samples *t*-test indicated that baseline SIS scores were not significantly different from each other between the two groups for any of the 8 SIS categories, nor for overall stroke recovery (Table 1). Groups did not differ in past medical history; however, 3 subjects noted health conditions (hypertension, hypercholesterolemia, and/or diabetes mellitus) for which no medications were listed. Overall the D-cycloserine + RAP treatment was generally safe and well tolerated. No serious adverse events occurred.

### 3.2. Behavioral Effects

Repeated measures ANOVA (between-subjects factor: treatment group (D-cycloserine + RAP, placebo + RAP); within subjects factor: time (pre- and posttreatment)) revealed significant effects of time. When average UE motor performance was examined, significant gains in grip strength were seen from baseline to week three for the affected hand, being 1.77 ± 4.14 newtons, 37.1% greater (*F*(1,12) = 19.839, *p* = 0.002, *η*_*p*_^2^ = 0.643; Table 2(a)). Changes in grip strength of the unaffected hand were not significant at follow-up. No significant changes were observed in the BBT of the affected or unaffected hand. When average cognitive measures were compared across all subjects, significant gains in reaction time were seen from baseline to week 3, being −425.81 ± 450.30 ms, 34.1% faster than at baseline (*F*(1,12) = 10.765, *p* = 0.014, *η*_*p*_^2^ = 0.495; Table 2(a)). Changes in sustained attention and working memory scores were not significant at follow-up. When average HRQL measures were compared, significant gains were seen in the SIS category evaluating mood and regulating emotion from baseline to week 3, being 8.97 ± 11.12, an 11.6% increase (*F*(1,12) = 7.653, *p* = 0.035, *η*_*p*_^2^ = 0.410; Table 2(a)). Overall changes in the remaining 7 SIS categories and stroke recovery were not significant (Table 2(a)).

Repeated measures ANOVA also revealed a significant main effect of treatment group for motor performance for grip strength (*F*(1,12) = 8.407, *p* = 0.026, *η*_*p*_^2^ = 0.433; Table 2(b)) and a significant time × treatment group interaction for the HRQL SIS stroke recovery domain (*F*(1,12) = 9.391, *p* = 0.020, *η*_*p*_^2^ = 0.461; Table 2(c)). No significant main effect of treatment group for the BBT of the affected or unaffected hand was observed.

To explore the effect of baseline performance on overall recovery, univariate ANCOVA (between-subjects factor: treatment group (D-cycloserine ± RAP, placebo ± RAP); covariate: baseline performance) was performed. The analysis revealed a significant main effect of group on SIS hand function domain (*F*(1,12) = 12.054, *p* = 0.013, *η*_*p*_^2^ = 0.573; Table 3(a)) only. The average change in affected hand use (SIS domain) was greater for the placebo + RAP (mean improvement 13.3 ± 23.2, 47.1% improvement; *F*(1,9) = 12.054, *p* = 0.013, *η*_*p*_^2^ = 0.573, Table 3(a)) compared with the D-cycloserine + RAP (mean improvement 10.0 ± 12.6, 41.2% improvement). No significant main effect of treatment group, time, or interaction was seen for the BBT or grip strength of the affected or unaffected hand.

ANCOVA also revealed a significant main effect of baseline performance of the CES-D (*F*(1,12) = 15.197, *p* = 0.007, *η*_*p*_^2^ = 0.628; Table 3(b)) and SIS categories for memory (*F*(1,9) = 19.591, *p* = 0.003, *η*_*p*_^2^ = 0.685), mood (*F*(1,9) = 8.484, *p* = 0.033, *η*_*p*_^2^ = 0.485), affected hand use (*F*(1,9) = 12.413, *p* = 0.011, *η*_*p*_^2^ = 0.580), and social participation (*F*(1,9) = 9.292, *p* = 0.026, *η*_*p*_^2^ = 0.508; Table 3(b)). Significant treatment group × baseline performance interaction was only observed for affected hand use (*F*(1,9) = 11.771, *p* = 0.013, *η*_*p*_^2^ = 0.567; Table 3(c)). A significant main effect of baseline performance on CES-D score improvement was also observed (*F*(1,9) = 15.197, *p* = 0.004, *η*_*p*_^2^ = 0.628; Table 3(b)).

To evaluate the potential clinical significance of the changes in average SIS Scores, odds ratio analysis was performed. For the D-cycloserine + RAP group, five of the nine SIS/stroke recovery scores improved by at least 10 points (which is considered to be a clinically significant improvement), while only one category improved by as much for the placebo + RAP group. While there was no significant association between treatment group and number of SIS categories that improved (odds ratio: 0.10, 95% CI: 0.452–221.080, *p* = 0.145), there was a strong degree of association between the treatment group and number of SIS categories that improved by the MCID: 4.5–17.8 points (*ϕ* ^2^ = 0.222, *p* = 0.036) [45]. Individual change scores were then collapsed across all SIS categories and positive versus neutral scores compared using a 2 × 2 OR table. Between the two groups, there was a significantly greater number of ≥4.5–17.8 change scores for the D-cycloserine + RAP group compared with placebo + RAP group, being 44.4% and 24.1%, respectively (odds ratio: 0.40, 95% CI: 1.063–5.988, *p* = 0.036), and a significant degree of association between the two variables (*ϕ* ^2^ = 0.045, *p* = 0.020).

### 3.3. Secondary Exploratory Analysis

In order to better understand the significant interaction between treatment group and SIS hand domain change score as determined by the ANCOVA, analysis of residuals was performed. D-Cycloserine + RAP had an average residual of 10 ± 2.0, while placebo + RAP had an average residual of 13.3 ± 20.4. However, upon comparing residuals of each participant, Tukey's schematic box plot analysis revealed that one participant in the control group had a significantly higher weighted score residual than the rest (mean residual: 7.92 ± 2.96) for both the balloon and thera-pong game. When this participant was removed from residual analysis, D-cycloserine + RAP had an average residual of 10 ± 2.0, while placebo + RAP had an average residual of 5 ± 0.0.

### 3.4. Robotic Weighted Score

**Figure 2:**
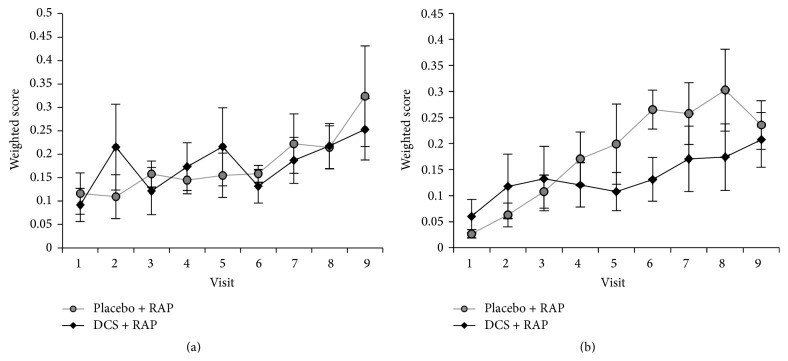
Robotic weighted score. Weighted scores on 6 times during which placebo or D-cycloserine (D-cycloserine) at 100 mg dose was dispensed. Across all subjects, mean weighted score on the (a) balloon game and (b) thera-pong improved significantly, from baseline to end of week 1, to week 2, and to week 3. However, there were no significant differences according to treatment group or interaction for any of these time intervals.

Nested repeated measures ANOVA (between-subjects factor: treatment group (D-cycloserine + RAP, placebo + RAP); two within-subjects factors: time (week, three levels) and treatment distribution (medication, no medication)) of the mean weighted balloon game score revealed a significant main effect of time across all subjects from baseline to week 3 (*F*(1,12) = 4.002, *p* = 0.033, *η*_*p*_^2^ = 0.267; Figure 2(a)).

There was also a significant main effect of time across all subjects for the mean weighted thera-pong score from baseline to week 3 (*F*(1,12) = 9.348, *p* = 0.001, *η*_*p*_^2^ = 0.459; Figure 2(b)), in addition to a main effect of treatment within each week (*F*(1,12) = 4.294, *p* = 0.027, *η*_*p*_^2^ = 0.281). There were no significant overall week × treatment group, treatment days × treatment group, or week × treatment day × treatment group interactions for either therapeutic training program. ANCOVA analysis was not performed as all patients began RAP on the same difficulty level.

## 4. Discussion

This preliminary study aimed to evaluate safety, motor, and quality-of-life effects of D-cycloserine + RAP in patients with chronic stroke. D-Cycloserine + RAP was safe and well tolerated. However, the main study hypothesis that D-cycloserine + RAP was superior to placebo + RAP for increasing upper limb motor function in adults with chronic stroke was not supported. At four weeks, there was no significant difference in primary or secondary outcomes. The only significant group effect uncorrected for any covariates was a relative improvement in grip strength of the affected hand. This effect did not remain significant after adjusting for baseline differences. The only significant group difference was observed in SIS hand function category for the affected hand in the RAP + D-cycloserine group.

Several lines of evidence suggest that activation of N-methyl-D-aspartate receptors (NMDA-R) is important for inducing various forms of synaptic plasticity that are critically involved in learning and memory. D-Cycloserine promotes NMDA receptor function by binding to receptors with unoccupied glycine sites and in addition perhaps by selectively enhancing the activity of NMDA-R [50] and might be useful for reversing deficits in function following stroke.

A recent study examined D-cycloserine + motor training as a potential enhancer of motor learning in able-bodied and stroke survivor participants [23]. Cherry et al. [23] found no significant difference in motor performance on a stability platform balance task (Lafayette Instrument, model 16030L) or a simulated feeding task [24], when a single dose of D-cycloserine (250 mg) was compared to placebo (250 mg) over the course of one training day. In addition, a single 250 mg dose of D-cycloserine failed to promote generalization of motor training on an untrained motor task [23]. The preliminary findings from Cherry et al. [23] evaluated the effects of one 250 mg dose of D-cycloserine on one day of motor training, in hopes of activating NMDA-R mediated LTP. However, unlike behavioral and psychiatric studies that have found efficacy in a single dose of D-cycloserine, motor learning may require repeated training exposure to enhance learning [24, 51]. In a recent randomized controlled trial provision of D-cycloserine (50 mg given each treatment day for up to ten weeks) failed to yield greater learning and retention of functional improvement achieved through therapy compared to placebo [25]. Our results support the finding of Nadeau et al. in that D-cycloserine in the dosing regime used plus repeated motor training sessions did not lead to enhanced motor learning compared to placebo.

There are several possible interpretations as to why no difference was observed between treatment arms on primary outcomes. The findings might indicate that increased NMDA-ergic tone simply does not engage procedure learning (i.e., those engaged in RAP) to the same extent as has been shown with implicit memory [21] and visuospatial learning [52] processes even though both engage NMDA-R dependent LTP mechanisms. Despite NR2C concentrations in hippocampal and neocortex interneurons [53, 54], human* in situ *mRNA probes reveal very weak signal density in the primary motor cortex [55]. Since the primary motor cortex plays an integral role in activity dependent LTP [56], very weak NR2C concentrations would limit the enhancing effects of D-cycloserine on NMDA-R dependent LTP. Although the specific mechanisms for RAP on NMDA-R dependent LTP remain unknown, it is possible that the RAP motor tasks selected for this study may have been NR2C containing and NMDA-R independent, subverting any potential beneficial effects of D-cycloserine.

Given that D-cycloserine can enhance certain models of plasticity, such as long-term potentiation [57], and has recently been investigated as an augmentation therapy for psychological treatment procedures [58], we might expect circulation NMDA-R to be associated with learning and memory. This possibility was indeed observed, with the partial Eta squared statistic indicating 37.6% of the variation in SIS memory domain and 57.3% of the variation in improvement in SIS hand function domain (Table 3(a)) could be attributed to the D-cycloserine group. Our observations are supported in that D-cycloserine has been shown to influence emotional learning, being a potentiator of extinction of conditioned fear in both animal models and human anxiety disorders [18, 59, 60].

While the molecular basis for the behavioral effects of D-cycloserine has not been elucidated, several clues exist as to why D-cycloserine might have unique behavioral action that other partial or full agonists at either the glycine or glutamate binding site on the NMDA receptor appear to lack. NMDA receptors are comprised of NR1, NR2A, and NR2B subunits, and D-cycloserine at maximally effective concentrations appears to cause slightly lower responses than maximally effective levels of glycine (the endogenous ligand) at NMDA receptors comprised of NR1/NR2A, NR1/NR2B, and NR1/NR2D subunits. D-Cycloserine causes current responses at NR1/NR2C receptors that are nearly twice as large as glycine [61]. That is, D-cycloserine appears to selectively enhance NMDA receptor function when the NR2C subunit is available. This fact suggests that the unique behavioral effects of D-cycloserine may be related to the potentiation of NR2C-containing NMDA receptors. Implicit in the hypotheses is the idea that enhancement of only NMDA receptors that contain the NR2C subunit may enhance emotional learning. In cortical structures (hippocampus and neocortex), NR2C subunit mRNA is expressed in subset of interneurons [53, 54] suggesting that modulation of NR2C function has the potential to sculpt network activity through modulation of interneuron firing. While the literature indicates there is a molecular basis for D-cycloserine to act as an enhancer through interneuron modulation [53, 54] and positive effect on extinction based learning in humans has been demonstrated [18, 60], our results indicate that D-cycloserine does not have the same beneficial effects on enhancing motor learning in our cohort of stroke survivors. Additional research in animals and humans is needed to clarify how D-cycloserine might enhance normal procedural learning.

The RAP intervention showed greater improvement in upper extremity motor function over the four-week period independent of treatment arm. RAP has emerged in the last decade and been promoted for restoring upper extremity motor function in stroke survivors [2, 62–64]. One interpretation of these results is that the action of repeatedly moving the wrist and hand during RAP was the primary stimulus to arm movement recovery for these participants. This hypothesis is consistent with other repetitive-movement exercise paradigms that improve upper extremity movement ability following brain injury [65–67]. Sensorimotor pathways become more reliable with repetitive activation as would be predicted by Hebbian learning [68]. Motor recovery seen in all volunteers was enhanced due to the active-assist nature of the robotic device. This observation corresponds well to the robotic studies that have successfully used external mechanical assistant to retrain arm ability after stroke.

Similar to other robot-assisted protocols [2], a major advantage of this study was that the active therapy protocol was controlled for intensity, duration, and method of movements and improved consistency and reproducibility of training. Both protocols included nine treatment sessions and a large number of repetitions per session. As has been suggested by others [2], the levels of improvement that were observed in the two therapy groups during the 3-week study suggest that high-intensity, repetitive, task-oriented movement training may be necessary for motor recovery. At present, we do not know whether a longer duration of therapy or more repetitions per session could lead to more meaningful results.

Between the two groups, there were a significantly greater number of ≥10 change scores on SIS categories for the D-cycloserine + RAP group compared with placebo + RAP group, being 44.4% and 24.1%, respectively (odds ratio: 0.40, 95% CI: 1.063–5.988, *p* = 0.036), and a significant degree of association between the two variables (*ϕ* ^2^ = 0.045, *p* = 0.020). People randomized to the D-cycloserine + RAP group were approximately 2.5 times as likely to exhibit a change score of ≥10 SIS categories compared to people randomized to the placebo + RAP group. Significant change in SIS hand function and stroke recovery scores have been shown in studies using a similar protocol [27]. A multicenter, randomized, controlled trial involving 127 patients with moderate to severe upper limb impairment 6 months or more after a stroke showed significant improvement in SIS categories with robot-assisted therapy as compared with usual care but found no significant benefit of robot-assisted therapy over intensive comparison therapy at 12 weeks on Fugl-Meyer or WMFT [2]. The lack of significant benefits of robot-assisted therapy on FM and WMFT was attributed to a cohort of patients with more severe impairments, who had multiple strokes and who were enrolled at longer period of times after stroke. Our participants were moderately impaired and were randomized approximately a year from index stroke compared to an average of 3.6 years from index stroke to randomization in study of Lo et al. [2].

Another interpretation as to why no significant treatment group differences were found may be that the preliminary study lacked sufficient power to detect any significant effects even if they exist in reality, which is reasonable given the small sample size of 14. Nonetheless, even a cursory look at the results indicates that moderately large differences exist between means, so utilizing the current result for power calculation of future studies appears to be justified. The partial Eta^2^ results indicate the relative degree with which the variance that was found in the ANOVA was associated with each of the main effects (group and visit) and their interaction. Partial Eta^2^ results can be interpreted as percentages of variance associated with each of the main effects, the interaction, and error. Interpretation of these partial Eta^2^ results indicated over 29.5% of improvement in grip strength of the affected hand, 27.5% improvement in memory, and 46.1% improvement in overall stroke recovery.

Many questions remain unanswered concerning the usefulness of D-cycloserine augmented physical therapy. For example, additional dose-finding research in animals and humans is needed to clarify how D-cycloserine might interact with other common medications. We did not exclude people who were on anticoagulant, cholesterol, hypertension, or diabetes therapy; and we did not screen antidepressant and benzodiazepine medications. The augmenting effect of D-cycloserine is potentially dependent on the timing and number of doses. Administration of D-cycloserine for a longer time (12 weeks versus 3 weeks) and using a longer duration between administration and initiation of physiotherapy (30 min versus 20 min) may lead to more meaningful outcomes. Further studies will be needed to determine optimal timing and duration of treatment, and it would need to be determined whether the treatment produced an effect above and beyond that achieved by robot-assisted physiotherapy alone.

## 5. Conclusion

Across all patients in this study, significant improvements in upper limb and other motor assessments were found over time. At doses achieved in this trial, D-cycloserine + RAP was generally safe and well tolerated; however, this combination did not show any improvement over and above the effects of placebo + RAP. Given the substantial evidence base demonstrating the role of NMDA receptor activity in learning and memory and our own positive preliminary findings, combining D-cycloserine with functional task practice may help reinforce the involved motor pathways at the synaptic level and thus enhance function performance over time.

## Figures and Tables

**Table 1:** Characteristics and baseline assessment of study participants as a whole and by group (^*∗*^note that mean DETECT *Z*-scores were computed for *n* = 6 and *n* = 5 for DCS + RAP and placebo + RAP groups, resp., due to missing values resulting from technical issues with the instrument). Hx: history; Tx: therapy; SIS: Stroke Impact Scale; TPA: tissue plasminogen activator.

	Total	D-Cycloserine + RAP	Placebo + RAP	*p*
	(*n* = 13)	(*n* = 7)	(*n* = 6)	
Age [mean (SD)]	55.8 (13.1)	57.7 (7.3)	53.7 (18.4)	0.608
Men/women	3/10	0/7	3/3	0.033
Caucasian/African American	3/10	1/6	2/4	0.459
Marital status [married/divorced/widowed/other]	6/2/1/4	4/0/1/2	2/2/0/2	0.814
Years of education completed	13.6 (4.0)	14.1 (2.1)	13.0 (5.6)	0.626
Days after stroke [mean (SD)]	391.2 (252.1)	456.6 (253.9)	315 (249.5)	0.334
Smoking Hx, >10 pack-years [yes, no]	1/12	1/5	1/6	0.915
Received TPA [yes, no]	4/9	3/4	1/5	0.349
Neurointerventional Tx [yes, no]	1/12	0/7	1/5	0.363
Baseline performance measures				
MMSE [mean score (SD)]	29.2 (1.1)	29.3 (0.8)	29.0 (1.5)	0.673
A-prime (SD)	0.9 (0.05)	0.9 (0.0)	0.9 (0.0)	0.085
Reaction time [sec (SD)]	1248.1 (700.9)	1147.8 (644.5)	1365.2 (806.2)	0.599
DETECT [mean *Z*-score (SD)]^*∗*^	10.1 (0.1)	10.2 (0.0)	10.1 (0.1)	0.393
Grip strength, affected hand [newtons (SD)]	4.8 (3.5)	4.0 (3.0)	5.7 (4.0)	0.390
Grip strength, unaffected hand [newtons (SD)]	28.6 (8.4)	23.6 (6.6)	34.5 (5.9)	0.011
BBT, affected hand [blocks moved (SD)]	9.7 (6.9)	9.7 (7.9)	9.6 (6.2)	0.991
BBT, unaffected hand [blocks moved (SD)]	45.9 (9.9)	48.7 (12.5)	42.7 (4.7)	0.289
CES-D [mean scale score, Day 1/mean (SD)]	12.3 (7.9)	15.1 (6.5)	9.2 (8.8)	0.185
SIS sections [scaled score (SD, FDR reported)]				
Strength	51.0 (14.4)	50.1 (13.2)	51.0 (17.0)	0.986
Memory	77.7 (18.3)	72.9 (19.7)	83.3 (16.5)	0.330
Mood	77.4 (15.4)	72.6 (16.3)	82.9 (13.5)	0.248
Communication	89.3 (16.8)	85.7 (22.3)	93.5 (6.2)	0.430
ADL/IADL	78.2 (17.2)	78.2 (19.9)	78.2 (15.2)	0.995
Mobility	70.0 (19.2)	64.6 (23.9)	76.3 (10.6)	0.297
Hand function	26.2 (21.2)	24.2 (29.6)	28.3 (4.1)	0.733
Social participation	62.7 (19.5)	53.6 (17.8)	73.4 (16.7)	0.064
Percent recovery	53.5 (9.9)	50.7 (9.3)	56.7 (10.3)	0.298
Previous diagnoses				
Hypertension [yes, no]	10/3	6/1	4/2	0.459
High cholesterol [yes, no]	7/6	4/3	3/3	0.817
Diabetes mellitus [yes, no]	3/10	1/6	2/4	0.459
Atrial fibrillation [yes, no]	0/13	0/7	0/6	—
Coronary artery disease [yes, no]	1/12	1/6	0/6	0.356
Prior stroke [yes, no]	1/12	1/6	0/6	0.356
Alcoholism [yes, no]	1/12	1/6	0/6	0.356
Substance abuse disorder [yes, no]	0/13	0/7	0/6	—
Medications				
Hypertension [yes, no]	8/5	6/1	2/4	0.059
High cholesterol [yes, no]	6/7	4/3	2/4	0.433
Diabetes mellitus [yes, no]	3/10	1/6	2/4	0.459
Anticoagulant [yes, no]	6/7	5/2	1/5	0.053

**Table:** (a) Repeated measures ANOVA (per visit) across all subjects

Source	*F*	*p*	*p*-RFDR	*η*_*p*_^2^
A-prime	2.852	0.119	0.225	0.206
Reaction time	10.765	0.007	0.014	0.495
DETECT *Z*-score	7.026	0.026	0.050	0.438
Grip strength, affected hand	19.839	0.001	0.002	0.643
Grip strength, unaffected hand	5.331	0.041	0.078	0.326
BBT, affected hand	0.022	0.884	1.670	0.002
BBT, unaffected hand	4.816	0.051	0.096	0.305
CES-D score	6.330	0.029	0.054	0.365
Stroke Impact Scale Category				
Strength	0.414	0.533	1.007	0.036
Memory	5.594	0.037	0.071	0.337
Mood	7.653	0.018	0.035	0.410
Communication	0.634	0.443	0.836	0.055
ADL/IADL	4.133	0.067	0.126	0.273
Mobility	0.649	0.438	0.826	0.056
Hand function	5.325	0.041	0.078	0.326
Participation	2.810	0.122	0.230	0.203
Stroke recovery	0.971	0.346	0.623	0.081

**Table:** (b) Repeated measures ANOVA (per group)

Source	*F*	*p*	*p*-RFDR	*η*_*p*_^2^
A-prime	2.424	0.148	0.279	0.181
Reaction time	0.316	0.586	1.106	0.028
DETECT *Z*-score	0.000	1.000	1.889	0.000
Grip strength, affected hand	1.493	0.247	0.467	0.120
Grip strength, unaffected hand	8.407	0.014	0.026	0.433
BBT, affected hand	0.043	0.840	1.587	0.004
BBT, unaffected hand	0.308	0.590	1.115	0.027
CES-D score	2.988	0.112	0.211	0.214
Stroke Impact Scale Category				
Strength	0.287	0.603	1.139	0.025
Memory	0.141	0.714	1.349	0.013
Mood	1.841	0.202	0.382	0.143
Communication	0.093	0.767	1.448	0.008
ADL/IADL	0.009	0.924	1.746	0.001
Mobility	0.691	0.424	0.800	0.059
Hand function	0.211	0.655	1.237	0.019
Participation	2.793	0.123	0.232	0.203
Stroke recovery	2.909	0.166	0.299	0.209

**Table:** (c) Repeated measures ANOVA (time × group interaction) across all subjects

Source	*F*	*p*	*p*-RFDR	*η*_*p*_^2^
A-prime	2.105	0.175	0.330	0.161
Reaction time	0.316	0.586	1.106	0.028
DETECT *Z*-score	0.633	0.447	0.844	0.066
Grip strength, affected hand	4.602	0.055	0.104	0.295
Grip strength, unaffected hand	2.060	0.179	0.338	0.158
BBT, affected hand	0.557	0.471	0.890	0.048
BBT, unaffected hand	2.585	0.136	0.257	0.190
CES-D score	0.216	0.651	1.230	0.019
Stroke Impact Scale Category				
Strength	0.259	0.621	1.172	0.023
Memory	4.177	0.066	0.124	0.275
Mood	0.206	0.659	1.245	0.018
Communication	2.925	0.115	0.218	0.210
ADL/IADL	0.164	0.693	1.309	0.015
Mobility	1.014	0.336	0.634	0.084
Hand function	0.109	0.748	1.413	0.010
Participation	1.779	0.209	0.395	0.139
Stroke recovery	9.391	0.011	0.020	0.461

**Table:** (a) ANCOVA (per group)

Source	*F*	*p*	*p*-RFDR	*η*_*p*_^2^
A-prime	0.003	0.959	1.812	0.000
Reaction time	0.041	0.844	1.594	0.005
DETECT *Z*-score	0.730	0.421	0.795	0.094
Grip strength, affected hand	0.829	0.386	0.730	0.084
Grip strength, unaffected hand	0.405	0.540	1.020	0.043
BBT, affected hand	0.111	0.747	1.410	0.012
BBT, unaffected hand	1.884	0.203	0.384	0.173
CES-D score	0.092	0.769	1.452	0.010
Stroke Impact Scale Category				
Strength	0.342	0.573	1.083	0.037
Memory	5.414	0.045	0.085	0.376
Mood	0.855	0.379	0.716	0.087
Communication	1.755	0.218	0.412	0.163
ADL/IADL	0.005	0.947	1.788	0.001
Mobility	0.536	0.483	0.912	0.056
Hand function	12.054	0.007	0.013	0.573
Participation	3.483	0.095	0.179	0.279
Stroke recovery	1.006	0.342	0.616	0.101

**Table:** (b) ANCOVA (per baseline) across all subjects

Source	*F*	*p*	*p*-RFDR	*η*_*p*_^2^
A-prime	0.978	0.348	0.658	0.098
Reaction time	6.427	0.032	0.060	0.417
DETECT *Z*-score	4.256	0.078	0.147	0.378
Grip strength, affected hand	1.487	0.254	0.479	0.142
Grip strength, unaffected hand	1.541	0.246	0.464	0.146
BBT, affected hand	0.001	0.975	1.842	0.000
BBT, unaffected hand	5.576	0.043	0.080	0.383
CES-D score	15.197	0.004	0.007	0.628
Stroke Impact Scale Category				
Strength	5.803	0.039	0.074	0.392
Memory	19.591	0.002	0.003	0.685
Mood	8.484	0.017	0.033	0.485
Communication	0.971	0.350	0.662	0.097
ADL/IADL	4.477	0.063	0.120	0.332
Mobility	0.934	0.359	0.678	0.094
Hand function	12.413	0.006	0.011	0.580
Participation	9.292	0.014	0.026	0.508
Stroke recovery	5.645	0.041	0.074	0.385

**Table:** (c) ANCOVA (group *∗* baseline interaction)

Source	*F*	*p*	*p*-RFDR	*η*_*p*_^2^
A-prime	0.001	0.980	1.852	0.000
Reaction time	0.024	0.879	1.661	0.003
DETECT *Z*-score	0.725	0.423	0.798	0.094
Grip strength, affected hand	0.009	0.926	1.750	0.001
Grip strength, unaffected hand	0.381	0.553	1.044	0.041
BBT, affected hand	0.724	0.417	0.787	0.074
BBT, unaffected hand	1.531	0.247	0.467	0.145
CES-D score	0.835	0.385	0.726	0.085
Stroke Impact Scale Category				
Strength	0.202	0.663	1.253	0.022
Memory	3.667	0.088	0.166	0.290
Mood	1.082	0.325	0.615	0.107
Communication	1.423	0.263	0.498	0.136
ADL/IADL	0.025	0.877	1.656	0.003
Mobility	0.450	0.519	0.981	0.048
Hand function	11.771	0.007	0.013	0.567
Participation	3.882	0.080	0.151	0.301
Stroke recovery	0.302	0.596	1.073	0.032
